# Greater Fatigue and Reduced Neurocognitive Speed With Symptomatic Crohn’s Disease

**DOI:** 10.1093/crocol/otae069

**Published:** 2024-12-23

**Authors:** Brittaney Bonhomme, Neilanjan Nandi, Shivali Berera, Helen Lee, Galen Leung, Chung Sang Tse, Alexandra Weiss, Lisa Nessel, Yue Ren, Hongzhe Li, Faten N Aberra, James D Lewis

**Affiliations:** Center for Clinical Epidemiology and Biostatistics, Perelman School of Medicine, University of Pennsylvania, Philadelphia, PA, USA; Division of Gastroenterology and Hepatology, Perelman School of Medicine, University of Pennsylvania, Philadelphia, PA, USA; Division of Gastroenterology and Hepatology, Perelman School of Medicine, University of Pennsylvania, Philadelphia, PA, USA; Division of Gastroenterology and Hepatology, Perelman School of Medicine, University of Pennsylvania, Philadelphia, PA, USA; Division of Gastroenterology and Hepatology, Perelman School of Medicine, University of Pennsylvania, Philadelphia, PA, USA; Division of Gastroenterology and Hepatology, Perelman School of Medicine, University of Pennsylvania, Philadelphia, PA, USA; Division of Gastroenterology and Hepatology, Perelman School of Medicine, University of Pennsylvania, Philadelphia, PA, USA; Center for Clinical Epidemiology and Biostatistics, Perelman School of Medicine, University of Pennsylvania, Philadelphia, PA, USA; Department of Biostatistics, Epidemiology and Informatics, Perelman School of Medicine, University of Pennsylvania, Philadelphia, PA, USA; Center for Clinical Epidemiology and Biostatistics, Perelman School of Medicine, University of Pennsylvania, Philadelphia, PA, USA; Department of Biostatistics, Epidemiology and Informatics, Perelman School of Medicine, University of Pennsylvania, Philadelphia, PA, USA; Division of Gastroenterology and Hepatology, Perelman School of Medicine, University of Pennsylvania, Philadelphia, PA, USA; Center for Clinical Epidemiology and Biostatistics, Perelman School of Medicine, University of Pennsylvania, Philadelphia, PA, USA; Division of Gastroenterology and Hepatology, Perelman School of Medicine, University of Pennsylvania, Philadelphia, PA, USA; Department of Biostatistics, Epidemiology and Informatics, Perelman School of Medicine, University of Pennsylvania, Philadelphia, PA, USA

**Keywords:** cognitive function, Crohn’s disease, fatigue, memory

## Abstract

**Background:**

While patients with Crohn’s disease commonly report fatigue, an association of Crohn’s disease with mild neurocognitive impairment has also been suggested. This study investigated the relationship between Crohn’s disease activity, fatigue, and neurocognitive functioning.

**Methods:**

In this cross-sectional study, adults with Crohn’s disease (*n* = 25) and healthy controls (*n* = 26) completed the PROMIS Fatigue 7a form and Multidimensional Fatigue Inventory and neurocognitive testing across 6 domains. Symptomatic and endoscopic remission were assessed with a short Crohn’s Disease Activity Index and Simple Endoscopic Score for Crohn’s Disease. Linear regression adjusting for age and sex was used to compare fatigue and neurocognition among patients with Crohn’s disease versus controls and those with active Crohn’s disease versus those in remission.

**Results:**

Compared to controls, adults with Crohn’s disease reported greater overall and domain-specific fatigue (general, physical, and mental) (*P* < .05 for all comparisons). Patients in symptomatic remission had significantly less fatigue (*P* < .05). No differences were found in neurocognitive accuracy or speed between Crohn’s disease and controls. Disease activity was not associated with accuracy on neurocognitive testing; however, patients with symptomatic Crohn’s disease had longer correct response times for social cognition and episodic memory compared to asymptomatic patients (*P* < .05). Endoscopic disease activity was associated with longer correct response times for tasks linked to social cognition, episodic memory, and complex cognition (*P* < .05). These differences persisted after adjusting for fatigue.

**Conclusions:**

Patients with symptomatic Crohn’s disease experience greater fatigue and have slower response times on neurocognitive testing. However, fatigue does not appear to mediate the slower response times.

## Graphical Abstract

**Graphical Abstract:**
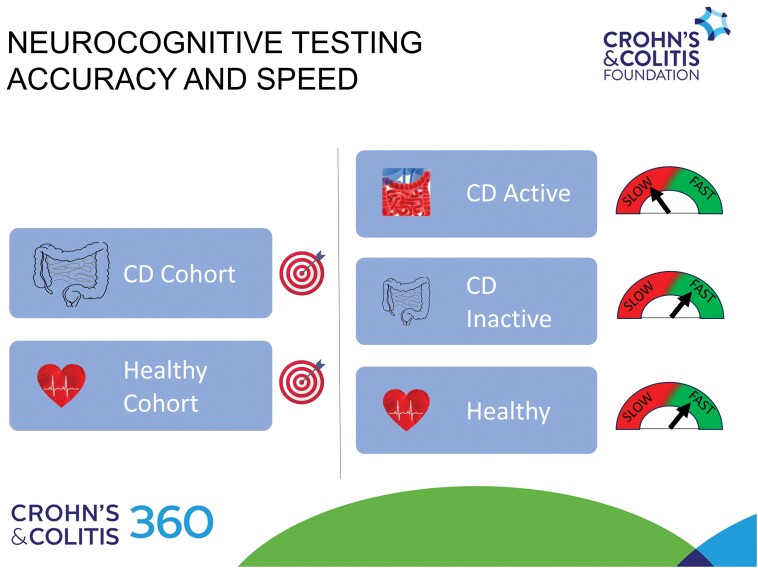

**Key Points**

- **What is already known?** While patients with Crohn’s disease commonly report fatigue, an association of Crohn’s disease with mild neurocognitive impairment has also been suggested.
- **What is new here?** In the Fatigue and Cognitive Testing in Crohn’s Disease study, Crohn’s disease activity was associated with slower performance but not reduced accuracy on neurocognitive testing and the slower performance was independent of fatigue.
- **How can this study help patient care?** These data justify accommodations for patients with active Crohn’s disease to support them in cognitively challenging activities.

## Background

Crohn’s disease is a chronic, immune-mediated inflammatory disorder of the digestive tract that affects approximately 1.5 million Americans.^1^ While it is characterized by variable periods of remission and activity, many patients are not fully asymptomatic even when in remission based on clinical markers. Other patients may have ongoing inflammation in the absence of gastrointestinal symptoms.

Fatigue, defined as self-reported tiredness or low energy, is more common among patients with Crohn’s disease than healthy controls, being reported in more than 50% of patients.^2–6^ Fatigue may manifest as physical or mental symptoms that impair quality of life and productivity. Relatively few patients have a resolution of fatigue over the short term.^7^

There are many causes of fatigue, one of which is poor-quality sleep. Poor sleep quality is common among patients with inflammatory bowel diseases (IBD), both those with active disease and in remission.^8–11^ Poor sleep quality is considered a risk factor or predictor of subclinical inflammation^12^ and future clinical relapse of Crohn’s disease.^13^

Neurocognitive function is conceptually related to fatigue, particularly mental fatigue. The neurocognitive function includes several different domains: executive function, episodic memory, complex reasoning, social cognition, visual-spatial, language, motor speed, and speed of processing.^14^ Neurocognitive testing can be done using a range of tools. More than 50 different tools have been employed to assess for mild cognitive decline.^15^ Some of the tools are commonly used in clinical practice, such as the Mini-Mental State Examination and Trail Making Test; others have been primarily used for research.

Impaired neurocognitive functioning could contribute to reduced academic and professional success and perhaps impaired safety when performing tasks that require rapid decision-making.^16–18^ Several small studies have observed that patients with Crohn’s disease have impaired cognitive function, particularly reduced response speed.^19–21^ A recent systematic review of neurocognitive testing in patients with IBD demonstrated reduced attention, working memory, and executive function compared to healthy controls.^22^

Most of the patients enrolled in the prior studies assessing neurocognitive function had quiescent Crohn’s disease, although a study by Golan et al.^19^ demonstrated an association of the Crohn’s Disease Activity Index with impaired neurocognitive functioning. We hypothesized that Crohn’s disease-associated fatigue may contribute to impaired neurocognitive functioning. In this study, we explored whether patients with Crohn’s disease have impaired neurocognitive functioning relative to people without Crohn’s disease and if disease activity and fatigue are related to impaired neurocognitive functioning.

## Methods

The Fatigue and Cognitive Testing in Crohn’s Disease (FACT CD) study was conducted at the University of Pennsylvania with enrollment completed between September 2022 and June 2023. FACT CD was a cross-sectional study of adults with and without Crohn’s disease. The Crohn’s disease cohort was recruited from patients undergoing a clinically indicated colonoscopy. The control cohort without Crohn’s disease consisted of people scheduled for a colonoscopy for colorectal cancer screening and a local listing of people interested in participating in clinical research but who were not scheduled to undergo colonoscopy.

All participants were between the ages of 30 and 65 years. Patients with a history of stroke, attention deficit hyperactivity disorder, dementia or cognitive disorder, cirrhosis, or active narcotic use other than diphenoxylate-atropine were excluded at the time of screening.

All enrolled patients completed patient-reported outcome measures and neurocognitive testing prior to their colonoscopy or between 2 and 14 days after the colonoscopy to avoid any influence of the anesthesia used during the procedure. Blood samples were collected immediately prior to the colonoscopy only from the Crohn’s disease cohort. Participants received a small financial compensation for participating in the study.

### Assessments

Neurocognitive testing was conducted using the Penn Computerized Neurocognitive Battery (CNB) which is a rigorously validated series of tests administered electronically and remotely to assess multiple domains of neurocognitive function.^14,23^ The CNB is administered in person or remotely using a personal computer. For this study, we used a remote application. The CNB requires approximately 1 hour to complete and includes 4 tests of executive function, 3 of episodic memory, 3 of complex reasoning, 2 of social cognition, 2 of motor speed, and 1 of the speed of processing (Table 1).^14,24–27,29–31^

**Table 1.** Neurocognitive tests by domain.

Domain	Test
Executive functioning	Abstraction and Mental Flexibility—Penn Conditional Exclusion Test—Subjects decide which of 4 objects does not belong with the other 3 based on one of three sorting principles^24^
	Attention—Continuous Performance Test—Number and Letter—Participant responds to a set of 7-segment displays presented 1/s., whenever they form a digit (NUMBERS, initial 3 minutes) or letter (LETTERS, next 3 minutes)^25^
	Working Memory—Letter-N_Back—presents letters for 500 ms, and the participant has an additional 2000 ms to respond by pressing the spacebar. There are three conditions: 0-Back—press the spacebar when the letter presented is an “X”; 1-Back—press when the letter presented is the same as the previous letter; 2-Back—press when the letter presented is the same as the one just before the previous letter^26^
	Inhibition—Go-No-Go Test—participants see Xs and Ys quickly displayed (300 ms) at different positions on the screen and are instructed to respond if and only if an X appears in the upper half of the screen, inhibiting the impulse to respond to Xs in the lower half of the screen and Ys generally^27^
Episodic memory	Facial Memory—presents 20 digitized faces that are then mixed with 20 distractors equated for age, gender, and ethnicity^28^
	Verbal Memory—presents 20 target words that are then mixed with 20 distractors equated for frequency, length, concreteness, and low imageability^29^
	Spatial Memory—uses Euclidean shapes as stimuli with the same paradigm as the word and face^23^
Complex reasoning	Penn Verbal Reasoning Test—multiple-choice test in which participants answer verbal analogy problems^14^
	Penn Matrix Analysis Test—consists of matrices requiring reasoning by geometric analogy and contrast principles^26^
	Penn Line Orientation Test—presents two lines at an angle, and participants mouse-click on a “button” that makes one line rotate until they consider it to have the same angle as the other^30^
Social cognition	Measured Emotion Differentiation Test—presents pairs of emotional expressions, each pair from the same individual expressing the same emotion, one more intense than the other or of equal intensity. The task is to click on the face that displays the more intense expression or indicate that they have equal intensity^14^
	Age Differentiation Test—requires the participant to select which of the two presented faces appears older, or if they are the same age^14^
Motor speed	Motor Praxis Test—requires moving the mouse and clicking on a green square that disappears after the click^14^
	Computerized Finger Tapping Test—measures how many times a participant can press the spacebar using only the index finger in 10 seconds^14^
Speed of processing	Digit Symbol Test—1 of 9 symbols appears on the screen paired with a number, and the participant decides whether the pairing is correct or incorrect^31^

Fatigue was assessed using two measures: the Patient Reported Outcomes Measurement Information System Fatigue 7a (PROMIS-F) and Multidimensional Fatigue Inventory (MFI). PROMIS-F assesses a range of self-reported symptoms from the prior week, ranging from mild subjective feelings of tiredness to an overwhelming, debilitating, and sustained sense of exhaustion that impacts the ability to execute daily activities and function in family or social roles.^32^ Response options for PROMIS-F measures are on a 5-point Likert scale, ranging from never to always. Responses are converted to *T*-scores, where 50 is the mean for the US population with a standard deviation (SD) of 10. The MFI is a 20-question scale covering 5 domains of fatigue: general, physical, mental, reduced motivation, and reduced activity. Questions are answered on a 5-point Likert scale of how the person has been feeling “lately,” with higher scores reflecting greater fatigue.

The PROMIS Emotional Distress Short Form consists of 15 items, 8 items on depression (Depression—Short Form 8a), and 7 items on anxiety (Anxiety—Short Form 7a). A higher score on these PROMIS short forms connotes more emotional distress (depression or anxiety). With a standardized normative *T*-score of 50 and an SD of ±10, *T*-scores <55 are considered normal, 55-60 mild, 60-70 moderate, and ≥70 severe distress.^33^

The PROMIS Sleep Disturbance Questionnaire (v. 1.0; 8a) consists of 8 items measuring self-reported perceptions of sleep quality, depth, and restoration within the past 7 days. This includes perceived difficulties falling asleep and staying asleep, as well as sleep satisfaction. Higher scores indicate greater sleep disturbance.

The short Crohn’s Disease Activity Index (sCDAI) is a self-reported survey that asks participants to rate their general well-being, abdominal pain, and the number of liquid or soft stools in the previous day. Higher scores reflect greater symptom burden with scores <150 representing symptomatic remission. The measure was originally designed using a 1-week diary,^34^ but a 1-day report, which was used for our study, has been previously demonstrated to correlate well with the weeklong measurement.^35^

Endoscopic disease activity among patients with Crohn’s disease was based on the Simple Endoscopic Score for Crohn’s Disease (SES-CD).^36^ SES-CD scores range from 0 to 56 with scores <3 considered quiescent.

Blood samples were collected for hemoglobin, ferritin, vitamin B12, thyroid stimulating hormone, and high sensitivity C reactive peptide (hsCRP) concentration and measured in the hospital’s clinical laboratory.

### Statistical methods

Characteristics of the study cohorts were compared using descriptive statistics and the Student’s *t*-test for continuous variables and Fischer’s exact test for categorical variables. Associations of fatigue and Crohn’s disease were assessed using linear regression adjusted for age and sex. Neurocognitive testing results were log-transformed and then converted to *z* scores based on the study cohort using the formula (*x −* mean(*x*))/SD(*x*) where SD is the standard deviation. The *z* scores for tests within the same domain were then averaged to obtain a single *z* score per domain. For example, for each individual, the *z* score for the 4 tests of executive function was averaged to give an overall *z* score for executive function. The association of Crohn’s disease with each domain was tested using linear regression with adjustment for age and sex and then adjusted for age, sex, and fatigue *T*-score from the PROMIS measure. This was then repeated within the Crohn’s disease cohort comparing those with and without symptomatic remission, endoscopic remission, and normal hsCRP. Analyses were done separately for accuracy, speed for correct answers, and speed for incorrect answers. Exploratory analyses of specific Crohn’s disease symptoms and patient-reported sleep interference were conducted in a similar manner.

A sample size of 25 per group provided 90% power to detect a difference of 0.94 units of SD between the groups. Assuming that one-third of patients with Crohn’s disease would have active disease, with 25 patients with Crohn’s disease, there would be approximately 80% power to detect a difference of 1.27 SD units.

## Results

The cohort included 25 patients with Crohn’s disease and 26 control subjects (Table 2). The mean age was comparable (42.7 years in Crohn’s disease and 42.2 years in controls). There were slightly fewer females in the Crohn’s disease group (68% vs 84%). Most cohort members had a bachelor’s degree or higher level of education. In the Crohn’s disease cohort, 48% of patients had nonstricturing and nonpenetrating behavior and 40% had a history of perianal disease. Most patients (88%) were receiving a biologic medication. The mean sCDAI was 133.6 (SD 73.6). Compared to controls, patients with Crohn’s disease had similar education and symptoms of depression and anxiety, but greater reported sleep interruption.

**Table 2.** Characteristics of the study cohort.

	Crohn’s disease (*n* = 25)	Control (*n* = 26)	*P* value
Age (mean y, SD) (range)	42.7 (9.9) (30-65)	42.2 (9.0) (30-58)	.84
Female sex	17 (68%)	21 (84%)	.32
Race			.74
Asian	1 (4%)	1(4%)	
Black	2 (8%)	5 (19%)	
White	21 (84%)	18 (69%)	
Preferred not to answer or missing	1 (4%)	2 (8%)	
Ethnicity			.32
Hispanic	0 (0%)	3 (12%)	
Not Hispanic	25 (96%)	21 (81%)	
Prefer not to answer or missing	1 (4%)	2 (8%)	
Education			.13
High school or less	1 (4%)	1 (4%)	
Some college	3 (12%)	5 (19%)	
4-year college degree	8 (32%)	5 (19%)	
Graduate school	9 (36%)	15 (58%)	
Unreported	4 (16%)	0 (%)	
Phenotype			
Inflammatory	12 (48%)	N/A	N/A
Stricturing	8 (32%)	N/A	N/A
Penetrating	5 (20%)	N/A	N/A
Perianal fistula (ever)	10 (40%)	N/A	N/A
Prior bowel resections (median, range)	0 (0-3)	N/A	N/A
Current medications			
5ASA	4 (16%)	N/A	N/A
Oral steroids	3 (12%)	N/A	N/A
AZA/6MP/MTX	8 (32%)	N/A	N/A
Anti-TNF	11 (44%)	N/A	N/A
Vedolizumab	2(8%)	N/A	N/A
Ustekinumab	9 (36%)	N/A	N/A
JAKi	0 (0%)	N/A	N/A
Short CDAI	133.6 (73.6) (58-324)	N/A	N/A
sCDAI >150	10 (40%)	N/A	N/A
SES-CD	1.6 (2.4) (0-9)	N/A	N/A
SES-CD >2	7 (28%)	N/A	N/A
hsCRP (mean, SD)	3.8 (4.4)	N/A	N/A
PROMIS Fatigue	52.1 (10.3)	49.9 (8.5)	.41
Promise Fatigue *T* ≥ 60	7 (28%)	3 (11.5%)	.17
MDFI Total	53.5 (3.3)	45.9 (3.1)	.11
PROMIS Anxiety *T*-score	50.51 (7.84)	49.37(6.51)	.57
PROMIS Depression *T*-score	47.68 (7.70)	48.11(7.53)	.84
PROMIS Sleep Disturbance *T*-score	54.12 (7.42)	47.97(8.50)	.008
Alcohol use (current use)	7 (28%)	7 (27%)	>.99
Tobacco use (current use)	0 (0%)	4 (15%)	.11
Recreational drugs (current use)	2 (8%)	0 (0%)	.24
Narcotic use (current use)^e^	1 (4%)	0 (0%)	.49
Anemia^a^ (*n*, %)	6 (24%)	N/A	N/A
Vitamin B12 deficiency^b^ (*n*, %)	0 (0%)	N/A	N/A
Iron deficiency^c^ (*n*, %)	6 (24%)	N/A	N/A
Abnormal TSH^d^ (*n*, %)	3 (12%)	N/A	N/A

^a^Hemoglobin concentration <12 g/dL in females or <13.5 g/dL in males.

^b^Vitamin B12 concentration <180 pg/mL.

^c^Ferritin concentration <20 ng/mL.

^d^Thyroid stimulating hormone <0.45 μlU/mL or >5.33 μlU/mL.

^e^One patient was taking Tramadol after a recent surgery.

### Fatigue

**Figure 1.**
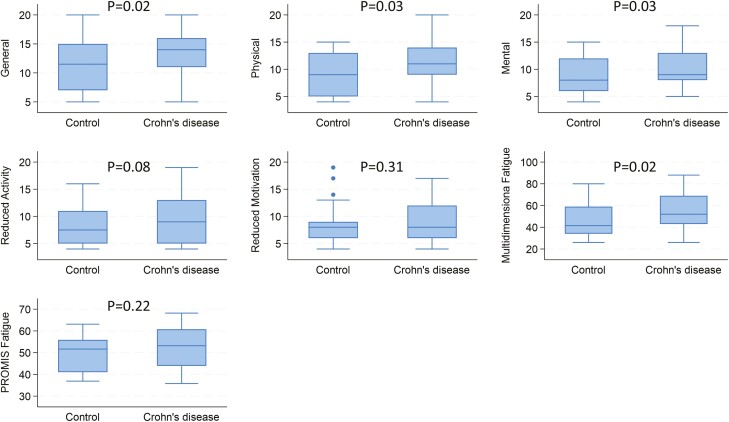
Association of fatigue and Crohn’s disease. *P* values are adjusted for age and sex.

Patients with Crohn’s disease were more likely to have experienced fatigue than control subjects (Figure 1). These patients reported greater overall fatigue as measured by the MFI total score (*P* = .02) and 3 of the domain scores: general (*P* = .02), physical (*P* = .03), and mental (*P* = .03). Although the MFSI total score and these three domains were all strongly correlated with PROMIS-F (Pearson *r*: 0.74 total, 0.83 general, 0.51 mental, and 0.59 physical), PROMIS-F was not significantly higher in patients with Crohn’s disease than controls (β 3.28, 95% CI −2.02, 8.58, *P* = .22).

Among those with Crohn’s disease, patients in symptomatic remission had significantly less fatigue on both overall measures (PROMIS Fatigue *P* = .01, MFI total *P* = .04) and 3 domain measures (general *P* = .01, physical *P* = .02, reduced motivation *P* = .02). However, neither endoscopic findings nor hsCRP were associated with any measures of fatigue (Table 3). In exploratory testing, anemia, iron deficiency, and abnormal TSH were not associated with any of the fatigue measures (Supplementary Table 1).

**Table 3.** Association of Crohn’s disease activity and fatigue.

	Symptomatic remission		Endoscopic remission		Normal hsCRP	
	Beta (95% CI)	*P* value	Beta (95% CI)	*P* value	Beta (95% CI)	*P* value
PROMIS F	−9.70 (−16.91 to −2.49)	.01	3.83 (−5.18 to 12.84)	.39	−.60 (−11.08 to 9.88)	.91
MFI Total	−12.82 (−25.29 to −0.35)	.04	7.86 (−6.66 to 22.39)	.27	−5.61 (−22.38 to 11.16)	.49
General fatigue	−3.52 (−6.21 to −0.83)	.01	2.72 (−0.44 to 5.89)	.09	−1.11 (−4.90 to 2.69)	.55
Mental fatigue	.04 (−3.40 to 3.48)	.98	1.45 (−2.23 to 5.13)	.42	−1.07 (−5.33 to 3.18)	.60
Physical fatigue	−3.56 (−6.57 to −0.55)	.02	1.27 (−2.40 to 4.94)	.48	−.66 (−4.83 to 3.51)	.74
Reduced activity	−1.45 (−4.41 to 1.51)	.32	2.08 (−1.07 to 5.24)	.18	−1.88 (−5.46 to 1.71)	.29
Reduced motivation	−4.33 (−7.91 to −0.76)	.02	.33 (−4.10 to 4.77)	.88	−.89 (−5.95 to 4.16)	.72

### Neurocognitive testing

**Figure 2.**
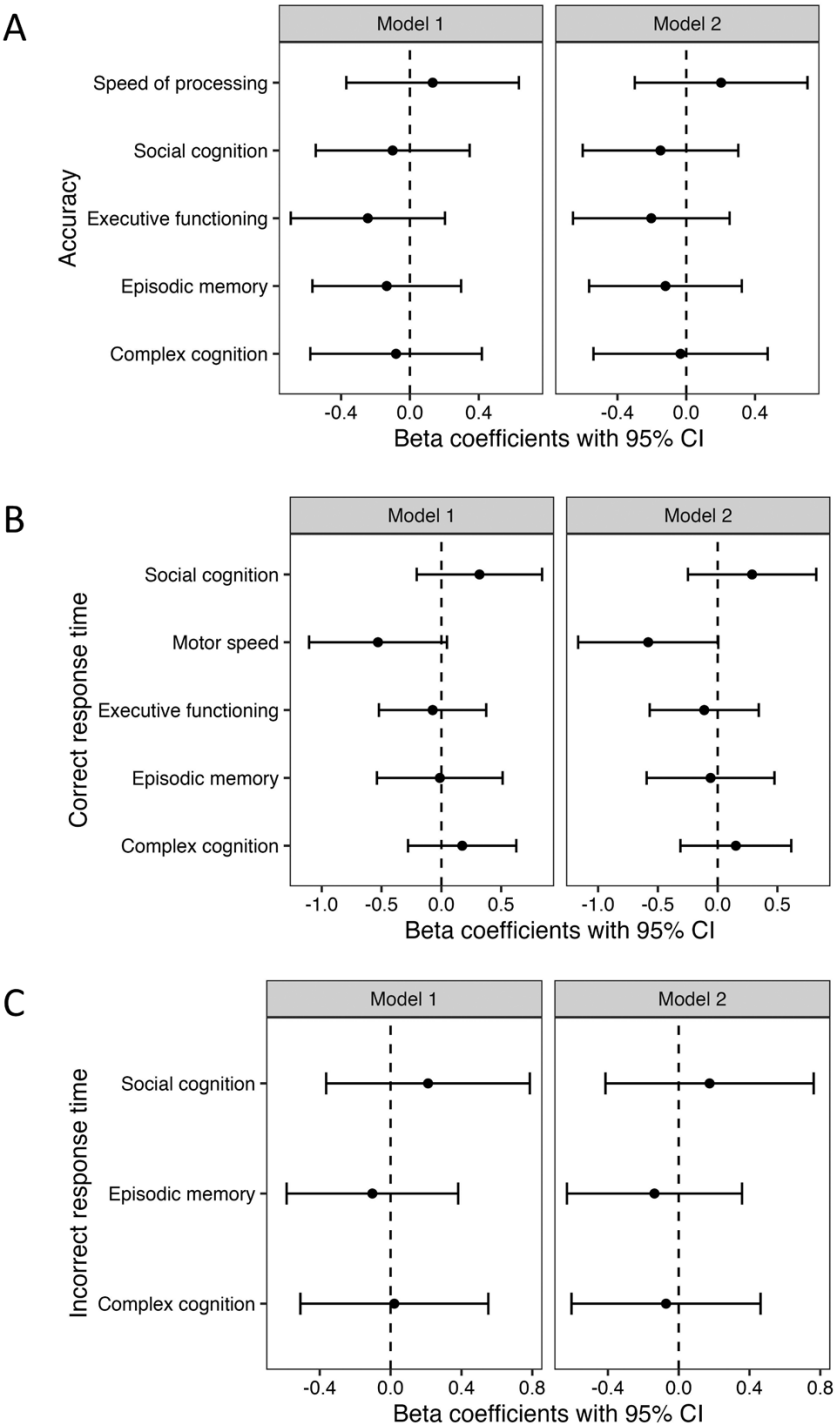
Association of Crohn’s disease and neurocognitive (A) accuracy, (B) correct response time, and (C) incorrect response time. Model 1 is adjusted for age and sex. Model 2 is adjusted for age, sex, and PROMIS Fatigue 7a *T*-score.

There were no significant differences in accuracy or speed between patients with Crohn’s disease and control subjects on all domains of the neurocognitive testing (Figure 2). Adjusting for fatigue had little impact on the results (Figure 2). Fatigue was not associated with accuracy or response time for any of the tests (*P* > .05 for all comparisons).

**Figure 3.**
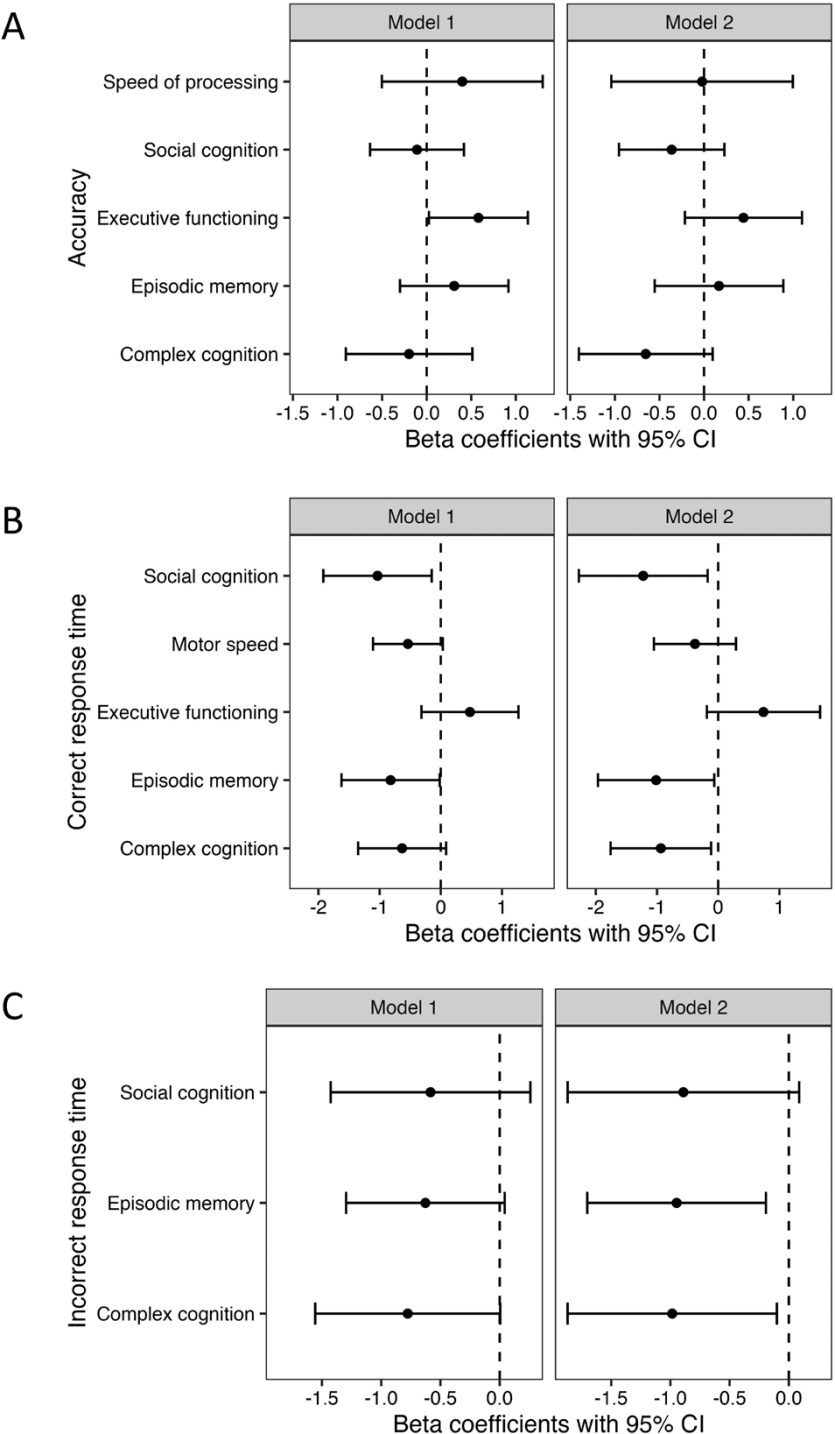
Association of Crohn’s disease symptoms and neurocognitive (A) accuracy, (B) correct response time, and (C) incorrect response time. Model 1 is adjusted for age and sex. Model 2 is adjusted for age, sex, and PROMIS Fatigue 7a *T*-score.

**Figure 4.**
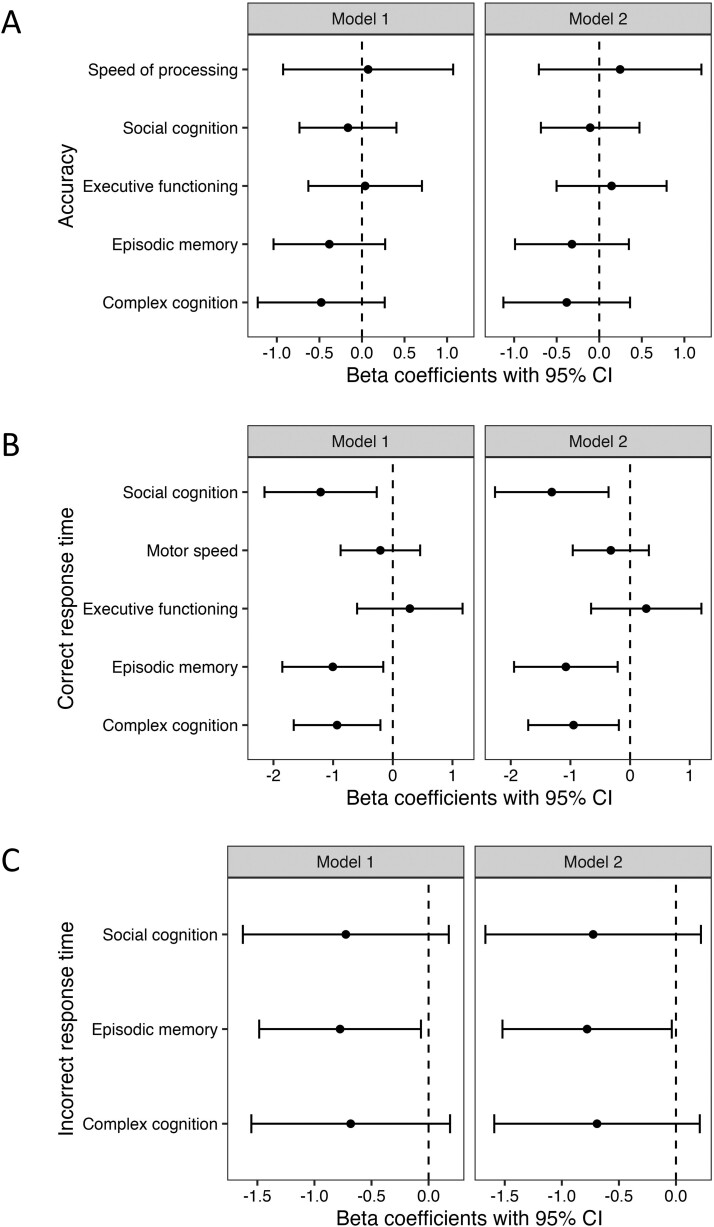
Association of Crohn’s disease endoscopic activity and neurocognitive (A) accuracy, (B) correct response time, and (C) incorrect response time. Model 1 is adjusted for age and sex. Model 2 is adjusted for age, sex, and PROMIS Fatigue 7a *T*-score.

Within the Crohn’s disease cohort, patients with symptomatic remission demonstrated higher accuracy for tasks categorized as executive functioning after adjusting for age and sex (β .58, 95% CI 0.03, 1.14, *P* = .03). With additional adjustment for fatigue, the association was attenuated and no longer statistically significant (β .44, 95% CI −0.21, 1.10, *P* = .18) (Figure 3A). Neither endoscopic remission (Figure 4A) nor normal hsCRP was associated with accuracy for any of the neurocognitive domains (Supplementary Figure 1).

Patients with symptomatic remission had shorter correct response times for all domains other than executive functioning in age and sex-adjusted models with differences in social cognition and episodic memory achieving statistical significance. While the association of symptomatic remission and motor speed was attenuated by adjustment for fatigue, the associations with correct response times for complex cognition, social cognition, and episodic memory were stronger after adjusting for fatigue (*P* < .05 for all comparisons) (Figure 3B). Similar results were observed for the speed of incorrect responses with episodic memory and complex cognition reaching statistical significance after adjusting for age, sex, and fatigue (Figure 3C).

Similar associations were observed for endoscopic assessment of disease activity. Patients with endoscopic remission had shorter correct response times for tasks linked to social cognition, episodic memory, and complex cognition in both models (Figure 4B) and incorrect response times for episodic memory (Figure 4C).

The concentration of hsCRP was not associated with the speed of correct or incorrect response times across any of the domains in both models (Supplementary Figure 1).

Because disease activity can be associated with mental health and mental health may impact neurocognitive function, a post hoc analysis examined the association of symptomatic remission and endoscopic assessment of disease activity with neurocognitive test performance adjusted for age, sex, and depression *T*-score. The results were nearly identical to the models adjusted only for age and sex. Significant associations (*P* < .05) with symptomatic remission were observed for social cognition and episodic memory correct response times and episodic memory incorrect response times. Significant associations (*P* < .05) with endoscopic disease activity were observed for complex cognition, social cognition, and episodic memory correct response times and episodic memory incorrect response times.

### Exploratory analysis of Crohn’s disease symptoms

Because the sCDAI, but not endoscopic remission nor hsCRP, was associated with fatigue, we conducted exploratory analyses of the different components of the sCDAI. The general well-being component of the sCDAI was the most strongly associated with fatigue (*P* < .03 for PROMIS-F and MFI Total) (Table 4). Abdominal pain was also associated with greater fatigue on the PROMIS-F scale (*P* = .03). In similar models of neurocognitive functioning, worse general well-being and greater frequency of loose bowel movements were associated with reduced accuracy on executive functioning tests (wellbeing β −.561, *P* = .01; stool frequency β −.251, *P* = .007) and slower response times on tests of motor speed performance (wellbeing β .536, *P* = .02; stool frequency β .218, *P* = .02). All other comparisons were not statistically significant. We also examine the correlation of sCDAI with depression and anxiety. There was a positive correlation, but this was not statistically significant when adjusted for age and sex (β 2.37, *P =* .36).

**Table 4.** Association of Crohn’s disease symptoms and fatigue.

	Stool frequency		Abdominal pain		General wellbeing	
	Beta (95% CI)	*P* value	Beta (95% CI)	*P* value	Beta (95% CI)	*P* value
PROMIS F	1.33 (−1.45 to 4.11)	.33	5.35 (0.44 to 10.27)	.03	9.62 (4.36 to 14.88)	.00
MFI Total	2.02 (−2.53 to 6.57)	.37	7.42 (−0.85 to 15.70)	.08	11.87 (2.11 to 21.62)	.02
General fatigue	.24 (−0.81 to 1.29)	.64	1.84 (−0.02 to 3.69)	.05	3.04 (0.92 to 5.16)	.01
Mental fatigue	.25 (−0.90 to 1.41)	.65	.50 (−1.72 to 2.72)	.64	.38 (−2.40 to 3.16)	.78
Physical fatigue	1.00 (−0.06 to 2.06)	.06	1.70 (−0.38 to 3.78)	.10	2.55 (0.04 to 5.06)	.05
Reduced activity	−.03 (−1.05 to 0.99)	.96	.27 (−1.69 to 2.24)	.78	1.38 (−0.99 to 3.75)	.24
Reduced motivation	.55 (−0.80 to 1.91)	.40	3.12 (0.88 to 5.36)	.01	4.51 (1.92 to 7.11)	.00

### Exploratory analysis of sleep interference

Patients with Crohn’s disease reported greater sleep interference and fatigue (Supplementary Table 2). We tested whether sleep interference and fatigue were associated with performance on neurocognitive accuracy and correct response time after adjusting for age, sex, and Crohn’s disease. PROMIS-F was not associated with accuracy or response time. Sleep interference was nominally associated with the speed of processing accuracy (*P* = .01) and with correct response time for episodic memory (*P* = .03) and motor speed (*P* = .01).

## Discussion

Fatigue is a widely recognized symptom of many chronic diseases, including Crohn’s disease. The impact of Crohn’s disease activity on neurocognitive function is less well studied but has been suggested in several small studies.^22^ In the FACT CD study, we demonstrated the expected association of Crohn’s disease with fatigue and demonstrated a novel association of Crohn’s disease activity with performance on neurocognitive testing that was largely independent of fatigue. While patients with Crohn’s disease had similar accuracy as healthy controls on most of the domains assessed, those with active Crohn’s disease symptoms or endoscopic activity demonstrated slower response times. This was evident in tests of social cognition, complex cognition, and episodic memory. These data support the concept that patients with Crohn’s disease can achieve comparable quality of cognitive work but may require additional time to complete tasks, particularly when their disease is active.

Investigation into the etiology of fatigue and impaired cognitive function in Crohn’s disease is in the early stages. Prior investigation has focused on the relationship of fatigue with clinical disease activity, systemic inflammation, microbiome and metabolome changes, anemia, diet quality, nutritional deficiencies, physical activity, seasonality (such as sun exposure, weather, etc.), sleep, and mental health.^10,37–41^ Additionally, in the general population, some evidence points to air quality influencing fatigue.^42–45^ Many of these factors are interrelated, although inflammation and bowel symptoms may be the central links.

We measured fatigue using a general and a domain-specific instrument. Patients with Crohn’s disease overall demonstrated increased physical and mental fatigue, but Crohn’s disease symptoms were only associated with physical fatigue. It is noteworthy that active Crohn’s symptoms were strongly associated with fatigue while endoscopic disease activity and elevated hsCRP were not. Moreover, the patient reported symptoms of reduced general well-being appeared to be more closely related to fatigue than abdominal pain or bowel frequency. The vast majority of patients in the Crohn’s disease cohort were treated with a biologic agent and one-third were receiving an immunomodulator medication. A meta-analysis of placebo-controlled trials in IBD demonstrated that biologic and newer small molecule medications improved fatigue relative to placebo.^28^ Nonetheless, it is possible that the medications used to treat active Crohn’s disease could have contributed to the association observed with fatigue.

Prior research in chronic pain, rheumatoid arthritis, heart failure, and other conditions has linked chronic disease with impaired neurocognitive function.^46–48^ The mechanisms by which chronic diseases impact cognitive function likely vary by underlying disease. We hypothesized that fatigue may mediate cognitive impairment in patients with Crohn’s disease. To test this, we assessed the association of Crohn’s disease and Crohn’s disease activity with neurocognitive test performance with and without adjustment for fatigue. Were our hypothesis correct, we would have expected to see attenuation of the associations with adjustment for fatigue. Although this was seen in a few examples, most of the associations with response times were independent of fatigue suggesting an alternative mechanism.

Numerous studies have used magnetic resonance imaging (MRI) to examine brain structure in patients with Crohn’s disease. Alterations have been reported in gray matter volume, subcortical gray matter volume, cortical surface area, and cortical folding.^49–54^ Reduction in gray matter volume has been particularly evident in the medial frontal gyrus which is associated with numeracy and literacy.^55^ In addition, patients with Crohn’s disease have been shown to have reduced cerebrospinal fluid volume which has been linked to cognitive impairment in the elderly.^56^ These findings are believed to reflect neuroplasticity in response to disease. The cross-sectional design of the prior studies limits the ability to assess the reversibility of these findings and their impact on fatigue and cognition. Longitudinal studies of disease activity, neuroimaging, and neurocognitive testing are needed to further clarify the relationship.

Neurocognitive tests are a surrogate for functioning ability in daily life. Prior studies have examined academic achievement in children with IBD, demonstrating poorer school attendance more so than poor academic performance.^57,58^ Studies in adults have demonstrated greater absenteeism from work in patients with Crohn’s disease, both before and after diagnosis.^59,60^ In a cross-sectional study of patients with IBD, self-reported difficulty concentrating, slow working pace, and delayed work production were the most commonly reported work-related difficulties.^61^ These features of presenteeism could be related to impaired neurocognitive functioning. Our results suggest that patients with Crohn’s disease do not have an appreciable decrease in the ability to correctly perform cognitive tasks but may require greater time. Thus, when needed, accommodations should be made in schools and the workplace.

The FACT CD had several important strengths. We utilized validated patient-reported outcome measures to assess fatigue. We used a battery of validated neurocognitive tests that allowed us to assess different domains of neurocognitive function both in terms of accuracy and response time. We used PROMIS measures for fatigue, sleep, and emotional distress. PROMIS measures are well-validated and allow for comparison to population norms.^62^ Moreover, PROMIS measures for fatigue have been demonstrated to be valid and responsive among patients with IBD.^63^ We included patients with both quiescent and active Crohn’s disease and included comprehensive measures of Crohn’s disease activity, including colonoscopy. We had a control cohort without Crohn’s disease who completed the same neurocognitive tests. Finally, we were able to test whether altered neurocognitive functioning in Crohn’s disease was mediated by fatigue.

A limitation of this study was the cross-sectional design which precluded us from measuring change in performance on neurocognitive testing or fatigue over time. This will be an important goal for future research. This study was relatively small and may have been underpowered to detect small differences. This also limited the number of covariates we could include in the regression models. Despite the small size, we demonstrated the ability to administer the neurocognitive tests remotely which supports the feasibility of conducting similar research on a larger scale. Because of the small size, we did not adjust for multiple comparisons. However, the number of tests meeting statistical significance at a nominal threshold of *P* < .05 was more than expected by chance and clustered together in the assessment of response time. We conducted 5 tests of accuracy across 4 main comparisons, none of which met nominal statistical significance. In contrast, 5 of 8 tests of response time were associated with symptomatic Crohn’s disease and 4 of 8 tests were associated with endoscopic inflammation using a nominal *P* value of .05 making it more likely that these are true associations.

Our cohort was highly educated, which may have impacted their performance. However, this was true of our control cohort as well. We also limited the cohort to those between the ages of 30 and 65 years since performance on neurocognitive testing tends to improve with age up to 30 and decline after 65. As such, our results may not be generalizable to the children, young adults, and elderly populations.

Patients with Crohn’s disease are more likely to experience mental health problems particularly when the disease is active.^64^ Although we did not observe a difference in depression or anxiety symptoms between the Crohn’s disease and control populations overall, the patients with active Crohn’s disease had greater depression and anxiety symptoms, although this was not statistically signifcant. Depression has been associated with reduced performance on neurocognitive tests^65^ but adjusting for depression *T*-score in this study had minimal impact on the results. Larger studies are needed to assess whether depression symptoms mediate the slower response times among patients with active Crohn’s disease.

In conclusion, in the FACT CD study, patients with Crohn’s disease did not differ from healthy controls on neurocognitive testing. Patients with active Crohn’s disease did not differ from those with quiescent disease in terms of neurocognitive testing accuracy, but they had slower response times. Despite the association of fatigue with Crohn’s disease symptoms, fatigue does not appear to mediate the slower response times among patients with active Crohn’s disease. These data may help clinicians understand and counsel patients with complaints of brain fog or other neurocognitive symptoms. Moreover, these data justify accommodations for patients with active Crohn’s disease to support them in cognitively challenging activities.

## Supplementary Material

otae069_suppl_Supplementary_Tables_S1-S2_Figure_S1

## Data Availability

Data from this study have not been deposited in a public repository. Requests for data should be made to the corresponding author.
