# Supplementary material for: Greater Fatigue and Reduced Neurocognitive Speed With Symptomatic Crohn’s Disease
Source: Crohns Colitis 360. 2024 Dec 23;7(1):otae069. doi: 10.1093/crocol/otae069 (PMC11684080; doi:10.1093/crocol/otae069)
Supplement: otae069_suppl_Supplementary_Tables_S1-S2_Figure_S1 [file otae069_suppl_supplementary_tables_s1-s2_figure_s1.pdf]

Supplemental Table 1. Association of blood test results and fatigue among patients with Crohn's disease

	Thyroid function		Anemia		Iron deficiency	
	Beta (95% CI)	P value	Beta (95% CI)	P value	Beta (95% CI)	P value
PROMIS F	-3.60 (-13.37 - 6.18)	0.45	-1.42 (-11.00 - 8.16)	0.76	0.82 (-9.41 - 11.05)	0.87
MFI Total	-8.43 (-24.12 - 7.26)	0.28	1.65 (-14.00 - 17.30)	0.83	3.50 (-13.10 - 20.10)	0.67
General fatigue	-1.63 (-5.22 - 1.96)	0.36	-0.41 (-3.97 - 3.15)	0.81	1.39 (-2.34 - 5.13)	0.45
Mental fatigue	-1.16 (-5.16 - 2.84)	0.55	0.09 (-3.83 - 4.01)	0.96	0.85 (-3.30 - 5.00)	0.67
Physical fatigue	-2.96 (-6.74 - 0.82)	0.12	1.62 (-2.19 - 5.44)	0.39	0.56 (-3.58 - 4.69)	0.78
Reduced activity	-0.19 (-3.75 - 3.37)	0.91	-0.54 (-3.92 - 2.84)	0.74	-0.01 (-3.68 - 3.66)	1.00
Reduced motivation	-2.49 (-7.14 - 2.16)	0.28	0.90 (-3.72 - 5.52)	0.69	0.71 (-4.23 - 5.64)	0.77

Supplemental Table 2. Association of sleep interference and fatigue with performance on neurocognitive tests

	Fatigue	Fatigue	Sleep	Sleep
Domain	Accuracy (β, P value)	Correct response time (β, P value)	Accuracy (β, P value)	Correct response time (β, P value)
Executive Functioning	-0.013 (0.32)	0.012 (0.34)	-0.003 (0.82)	0.025 (0.08)
Episodic memory	-0.004 (0.73)	0.014 (0.34)	-0.000 (0.99)	0.036 (0.03)
Complex reasoning	-0.015 (0.30)	0.007 (0.60)	0.004 (0.83)	0.006 (0.69)
Social cognition	0.015 (0.23)	0.009 (0.53)	0.010 (0.49)	0.010 (0.55)
Motor speed	N/A	0.01574 (0.33)	N/A	0.04547 (0.01)
Speed of processing	-0.022 (0.12)	N/A	-0.044 (0.005)	N/A

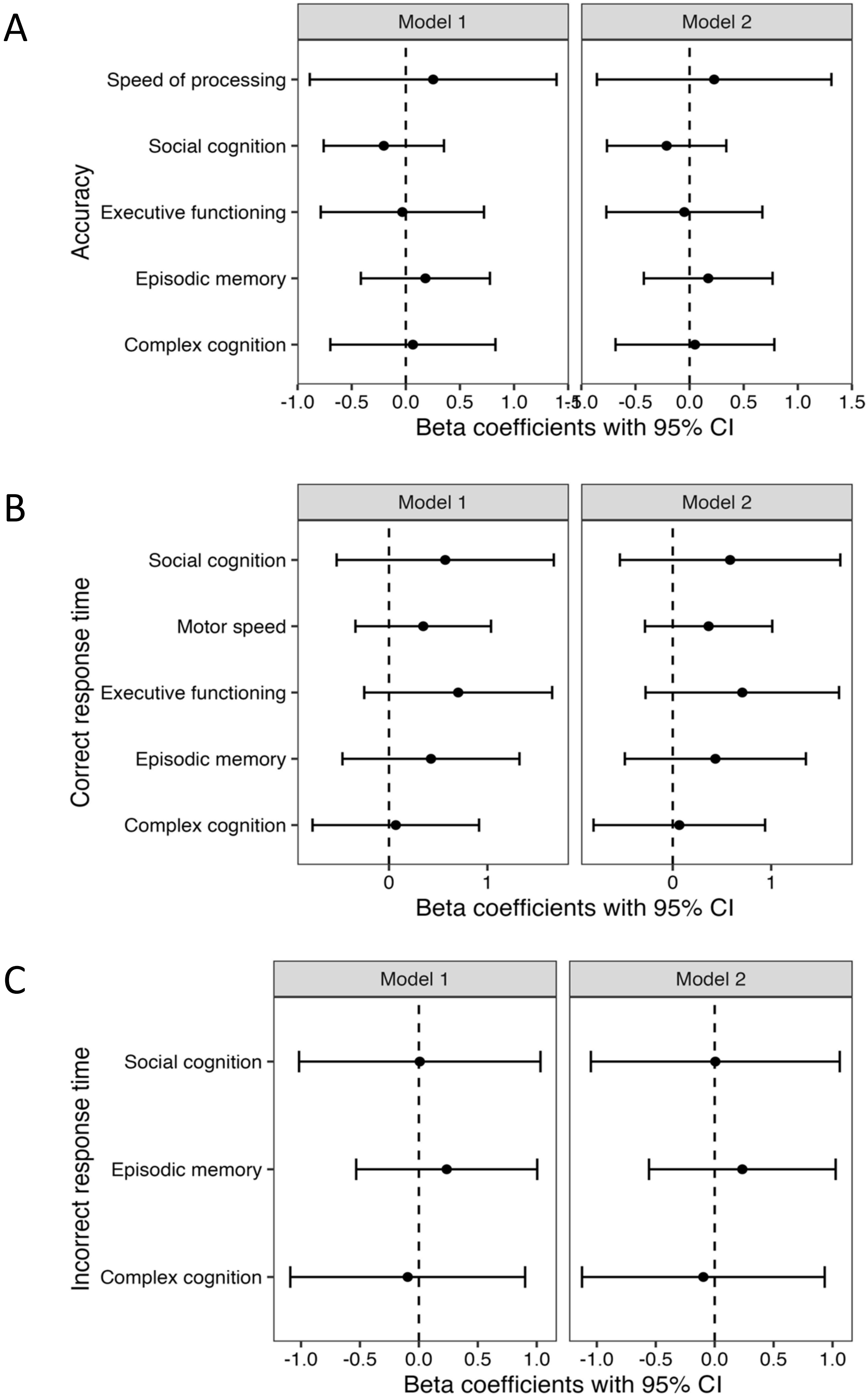

Supplemental Figure 1. Association of elevated high sensitivity CRP and neurocognitive (A) accuracy, (B) correct response time and (C) incorrect response time. Model 1 is adjusted for age and sex. Model 2 is adjusted for age, sex and PROMIS Fatigue 7a t score.
